# A Visual Compendium of Principal Modifications within the Nucleic Acid Sugar Phosphate Backbone

**DOI:** 10.3390/molecules29133025

**Published:** 2024-06-26

**Authors:** Daria Novikova, Aleksandra Sagaidak, Svetlana Vorona, Vyacheslav Tribulovich

**Affiliations:** Laboratory of Molecular Pharmacology, St. Petersburg State Institute of Technology, St. Petersburg 190013, Russia; aleksandrasagaidak@yandex.ru (A.S.); s.vorona@bk.ru (S.V.)

**Keywords:** artificial nucleic acid, antisense, oligonucleotide, chemical modification, synthetic biology

## Abstract

Nucleic acid chemistry is a huge research area that has received new impetus due to the recent explosive success of oligonucleotide therapy. In order for an oligonucleotide to become clinically effective, its monomeric parts are subjected to modifications. Although a large number of redesigned natural nucleic acids have been proposed in recent years, the vast majority of them are combinations of simple modifications proposed over the past 50 years. This review is devoted to the main modifications of the sugar phosphate backbone of natural nucleic acids known to date. Here, we propose a systematization of existing knowledge about modifications of nucleic acid monomers and an acceptable classification from the point of view of chemical logic. The visual representation is intended to inspire researchers to create a new type of modification or an original combination of known modifications that will produce unique oligonucleotides with valuable characteristics.

## 1. Introduction

Natural nucleic acids, DNA and RNA, form the basis of all living beings. These molecules are not only involved in the storage, transmission, and reproduction of information but also perform various, sometimes quite complex, functions from the catalysis of chemical reactions to the control of gene expression [1]. The formation of complex three-dimensional structures due to complementary interactions of nitrogenous bases is a fundamental property of these natural polymers. The prospect of influencing processes determined by this property gave impetus to the development of oligonucleotide chemistry, from its methodological aspects, synthetic schemes, and automated protocols to its wide range of applications.

The study on the structure and properties of nucleic acids allowed one to decode the human genome and understand the nature of many hereditary diseases. Oligonucleotide sequences capable of interacting with natural nucleic acids have enormous potential as means for studying biological systems and processes, diagnostic tools, and new biomedical products. However, the use of oligonucleotides based on natural nucleic acids has its own limitations, including insufficient resistance to enzymatic degradation in biological media caused by the action of nucleases that cleave phosphodiester bonds, low bioavailability, and the joint use of specific delivery systems [2]. To solve these problems and impart the required properties to polymers, the structural unit of natural nucleic acids is subjected to modification, and this subsequently led to the formation of a new direction of research [3].

Modified oligonucleotides, or artificial genetic polymers, have become an essential instrument in such therapeutic approaches as RNA interference [4], aptamer binding [5], and CpG oligonucleotide immunostimulation [6]. CRISPR/Cas9 genome editing has gained enormous popularity with the application of modified guide RNAs [7,8]. However, the most fruitful approach, which resulted in the first FDA-approved oligonucleotide drug, is the antisense oligonucleotide (ASO) principle [9]. While the therapeutic effect of ASOs is primarily associated with the cleavage of target mRNA by the RNase H enzyme, these short-strand nucleotide analogs can block mRNA processing or translation upon binding, form triple helices with DNA, prevent splicing or destabilize pre-mRNA, and attach to miRNAs, thus inhibiting transcription and affecting gene expression. The ASO strategy extensively recruits nucleic acid modifications to increase efficacy, enzymatic stability, and decrease the immune response and off-target toxicity of the developed oligoes [10].

This review is aimed at systematizing information about the currently known principal structural modifications of the sugar phosphate backbone of natural nucleic acids. These modifications, either alone or in combination, can impart artificial polymers with the desired properties and level out the drawbacks of oligonucleotide chains to provide wider experimental and medical applications.

## 2. Natural Nucleic Acids and Modification Directions

**Figure 1:**
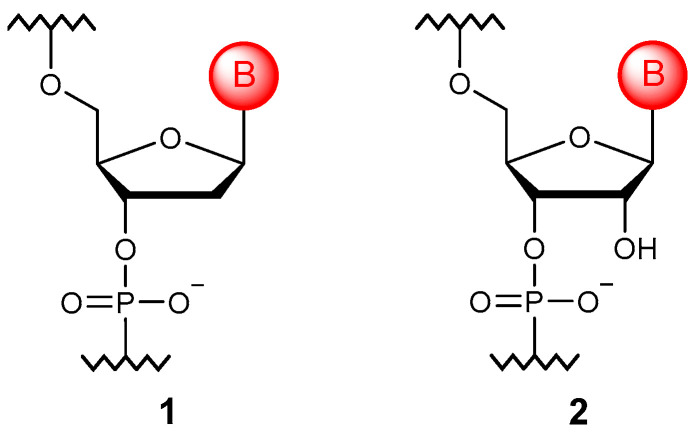
The structure of DNA (**1**) and RNA (**2**) monomer units.

Nucleic acids (NAs) are biological polymers whose monomer units are nucleotides (Figure 1). Nucleotides have a similar structure in both DNA (**1**) and RNA (**2**): a nitrogenous base, a pentose sugar, and a phosphate group. While the RNA monomer is based on β-D-ribose, the DNA chain is built on β-D-deoxyribose units linked by phosphodiester bonds. The pentose ring, through a glycosidic bond, carries one of five possible nitrogenous bases, with three (adenine, guanine, and cytosine) being common to DNA and RNA and the other two found only in a specific NA (thymine in DNA; uracil in RNA). These five nitrogenous bases are usually called major nucleobases since many naturally occurring modified ribonucleosides can be found in tRNA, rRNA, mRNA, and lncRNA molecules [11].

The finite set of nitrogenous bases, the instability under the action of nucleases, and specific physicochemical characteristics of natural nucleic acids have inspired researchers to search and develop analogs with certain properties that allow one to more fully utilize the inexhaustible potential of these unique biomolecules for both biomedical and research purposes. Over 50 years of intensive research in the field of oligonucleotide chemistry has made it possible not only to artificially synthesize natural nucleic acid oligomers but also to develop synthetic approaches for modifying their main structural elements. The proposed review is an attempt to systematize knowledge about the nucleotide analogs synthesized to date and identify and classify the basic modifications affecting the sugar and phosphate moieties in oligomers. This review does not discuss modified nitrogenous bases and their artificial analogues; achievements in this area of research can be found in the following papers [12,13].

## 3. Phosphate Modifications

The anionic nature and poor metabolic stability of the natural phosphodiester group are two main problems when using oligonucleotides as therapeutic agents. In this regard, modification of the phosphodiester group has historically become the first modification aimed at both increasing resistance to nucleases and overcoming biological barriers such as cell membranes. The types of modifications given below are used specifically for constructing oligomeric chains; however, there are also modifications that allow crosslinking oligomers to each other. Such modified NAs, such as borono-analogs [14], can formally be considered carrying a substitution for the phosphodiester bond, but the functional purpose of this modification is different.

### 3.1. Phosphotriesters

**Figure 2:**
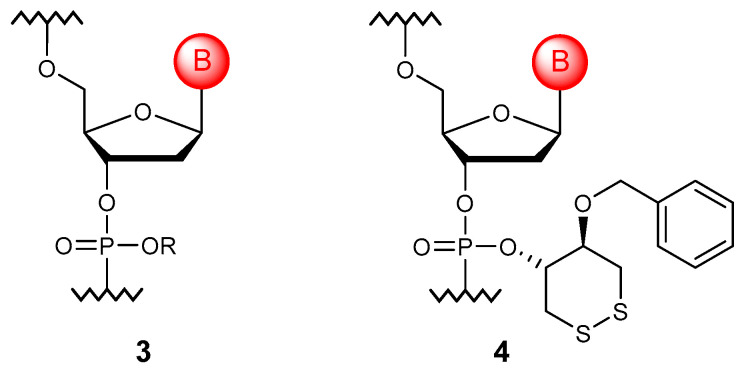
General structure of the phosphotriester monomer unit (**3**) and selected phosphotriester modification (**4**).

The main purpose of esterification (Figure 2) is to remove the negative charge from the nucleic acid backbone. It is believed that such derivatives (**3**) can hybridize with natural NAs, forming more stable duplexes due to the elimination of electrostatic repulsion [15]. To date, not only ethers, such as methyl, ethyl, isopropyl, and others [16,17], but ether modifications carrying functional groups [18], polyethylene glycol chains [19], fluorescent labels [20], and hydrophobic and cationic substituents [21] have been obtained. However, such a modification acquired its main practical significance in the context of delivering oligonucleotides into the cell: prodrug-type modifications (**4**) that impart not only resistance to the action of enzymes but also increase uptake by the cell were developed [22,23,24].

Interestingly, the phosphotriester modification can be obtained by reducing borane phosphonate oligonucleotides with metal ions [25]. In addition, the elementary units themselves can be used as esterification agents; thus, branched oligonucleotides could be obtained [26].

### 3.2. Substitution of Non-Bridging Oxygen Atoms

**Figure 3:**
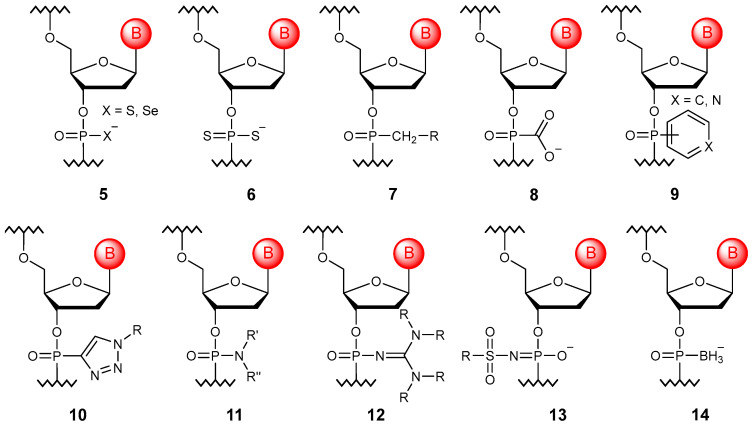
Structures of DNA monomer units with non-bridging oxygen modifications (**5**–**14**).

The non-bridging oxygen atom can be replaced with sulfur or selenium to obtain phosphorothioate [27] and selenoate [28] monomers (**5**), which allows oligonucleotides with such modifications to impart resistance to nucleases (Figure 3). Despite the frequent representation as =S, it was shown that sulfur does not form a double bond [29], so cationic oligonucleotides could be obtained [30]. Due to the retention of a negative charge on each internucleotide function, phosphorothioate derivatives are the closest analogues of natural oligonucleotides. However, such a substitution leads to chirality on phosphorus, and, therefore, approaches to the stereoselective synthesis of such derivatives have been developed [31,32].

If both non-bridging oxygens are replaced by sulfur, phosphorodithioate monomers (**6**) are obtained [33]. Such compounds are not chiral and are absolutely resistant to the action of all known nucleases. However, they are not widely used in the development of potential drugs since such a substitution results in the nonspecific binding to partially complementary DNA as well as to proteins. Most often, phosphorodithioate oligonucleotides are used in the design of aptamer libraries [34].

Another significant modification is the substitution of oxygen with carbon (**7**), primarily the methyl group (R = H) [35]. Oligonucleotides containing such nonionic bonds are resistant to degradation by cellular nucleases and are consumed by cells unchanged. At the same time, alkylphosphonates can be obtained enzymatically [36]. Alkyl can be used to attach some functional groups to phosphorus. So, to reduce the total negative charge, cationic units, such as alkylaminophosphonates [37,38], can be introduced into the oligomer structure. On the other hand, since the loss of the negative charge leads to poor solubility and the aggregation of oligomers in aqueous solutions, negatively charged modifications of non-bridging oxygen that remain resistant to nucleases were developed. The carboxyl group can be linked to phosphorus through a methylene bridge, such as in phosphonoacetates [39], or directly attached to the phosphorus atom to obtain phosphonoformates (**8**) [40]. In the form of neutral esters, these modified oligonucleotides are consumed by cells through passive diffusion.

In addition, other derivatives, such as phenylphosphonates and pyridylphosphonates (**9**) [41,42], as well as triazolylphosphonates (**10**), were synthesized. The latter are obtained from alkynylphosphonates via the click reaction [43]. The binding of phosphorus to the aromatic system makes oligonucleotides even more resistant to nucleases, probably due to their greater steric hindrance.

However, phosphoramidates (**11**) are the most widespread modified oligonucleotides. The non-bridging oxygen atom has been substituted with nitrogen to obtain various derivatives, starting from alkyl phosphoramidates [44], triazinyl phosphoramidates [45], and phosphoramidates with cationic substituents [46], including those containing guanidine groups [47]. Although phosphoryl guanidines (**12**) [48] are structurally close to phosphoramidates, the prospects of combining these oligonucleotides with phosphorothioate modifications for different therapeutic applications were repeatedly noted, while at least three stereo-regular mixed backbone phosphorothioate-phosphoryl guanidine oligonucleotides entered clinical trials in 2021 [49]. Another practically valuable group of modifications that should be mentioned here is substituted sulfonyl phosphoramidates (**13**), among which mesyl phosphoramidates (R = CH_3_) turned out to be promising for replacing phosphorothioate groups in ASOs [50]. Such modified oligonucleotides have shown significant advantages over commonly used phosphorothioates in their affinity to RNA, nuclease stability, and specificity of antisense action [51].

Separately, it is worth mentioning the boranophosphate modification (**14**) [52], which can be considered a functional hybrid of phosphate, phosphorothioate, and methylphosphonate fragments. In such oligonucleotides, the borane group is isoelectronic to O and S, and the internucleotide group is negatively charged like the phosphodiester group in native and phosphorothioate polymers. The boranophosphate group is also isostructural with the diester moiety of nuclease-resistant methylphosphonates. In addition, oligonucleotides modified in this way can be used as precursors to introduce other substitutions of one or both non-bridging oxygen atoms [53].

### 3.3. Substitution of Bridging Oxygen Atoms

**Figure 4:**
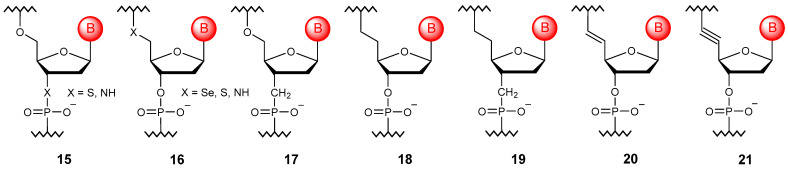
Structures of DNA monomer units with bridging oxygen modifications (**15**–**21**).

Another type of phosphate group modification is the replacement of bridging oxygens at positions 3′ (**15**) or 5′ (**16**) with selenium, sulfur, or nitrogen atoms to produce their corresponding selenophosphates [54], thiophosphates [55], and phosphoramidates [56,57]. The main advantage of such substitutions is an increase in the resistance to nucleases without the appearance of a chiral center (Figure 4). An interesting fact is that the unnatural 3′N–P bond can be formed naturally by a modified DNA polymerase [58].

One can also replace the oxygen atom with carbon. In this case, it is also possible to synthesize both 3′-methylenephosphonate (**17**) [59] and 5′-methylenephosphonate oligomers (**18**) [60], while the replacement of both atoms allows one to obtain bismethylenephosphinates (**19**) [61]. Other possible carbon substitutions of the bridging oxygen atom are represented by vinylphosphonate oligonucleotides (**20**), for which it is possible to vary the configuration due to E/Z isomerism [62], as well as alkynylphosphinate oligonucleotides (**21**), which allow one to improve the duplex-stabilizing properties due to the leveling of protons in position 6′ repelling the nitrogenous base [63]. However, formally, these substitutions already affect the sugar residue. 

In practice, it is quite rare to find only one modification; most often, combinations of bridged and non-bridged substitutions are used. The most common oligonucleotides are thiophosphoramidate ones [64].

### 3.4. Phosphate Linkage Extension

**Figure 5:**
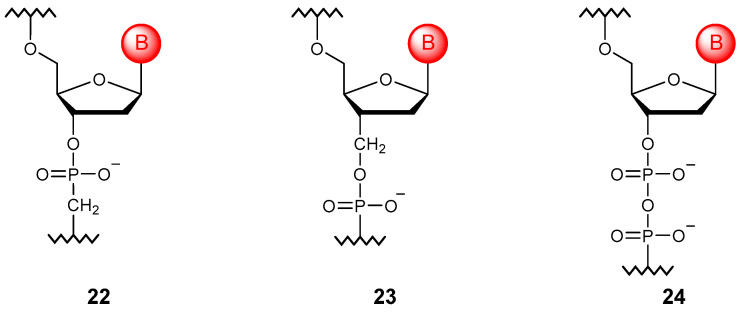
Structures of phosphate-extended DNA monomer units (**22**–**24**).

The phosphate unit can be elongated by adding the methylene group to the chain (Figure 5). In this way, it is possible to obtain methylene phosphonate derivatives (**22**) and (**23**), which have significant resistance to nucleases but retain the ability to form duplexes with natural NAs [65,66]. Another method of extension is to repeat the phosphoester unit. Such fully modified diphosphodiester oligomers (**24**) retain the ability to undergo complementary interactions and exhibit significant stability [67].

## 4. Sugar Linking Backbone Modifications

Modifications in this section affect both the phosphate group and the sugar residue. Oligonucleotides modified in this way most often do not retain their 3′ and 5′ oxygen atoms, the presence of which is determined by the structure of ribofuranose. However, it is generally accepted to consider changes of this kind as internucleotide linkage modifications.

A large number of works are devoted to the development and use of deoxyribose and ribose backbone cross-links that are alternatives to the phosphate group. When making such substitutions, they are primarily guided by the total length, which should be close to the native one to preserve the possibility of complementary interactions, as well as by the useful physicochemical parameters of the proposed crosslinking. All such modifications can be divided into acyclic and cyclic modifications.

### 4.1. Acyclic Linkages

**Figure 6:**
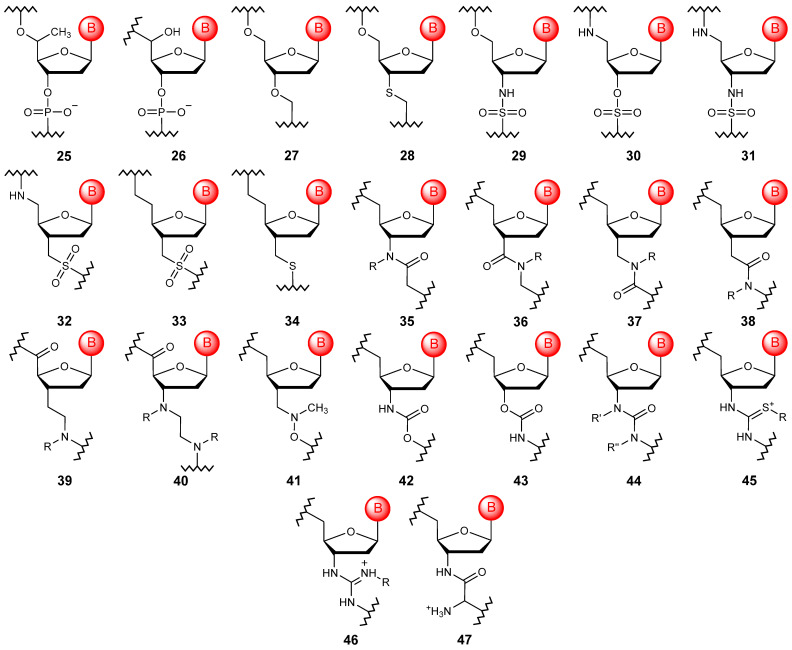
Structures of DNA monomer units with acyclic linkages (**25**–**47**).

Firstly, the introduction of a substituent to the carbon atom at position 5′ should be considered an alternative linkage (Figure 6). In particular, the introduction of the methyl substituent (**25**) allows one to obtain modified oligonucleotides with characteristics more suitable for use in antisense therapy, although it leads to the appearance of a chiral center on the carbon atom [68,69]. To date, the introduction of other substituents has been described, but their use is limited to terminal positions in small interfering RNAs [70]. Minimal modification of the natural phosphate linkage allows the production of α-hydroxyphosphonate oligonucleotides (**26**), which have marked differences in hybridization properties depending on the configuration of the 5′ carbon atom [71].

The formal replacement of the phosphorus atom and two non-bridging oxygen atoms with a carbon atom allows the formation of oligonucleotides with formacetal linkage (**27**) [72]. Such a minor structural change can be easily introduced into the polymer without compromising the helical structure and the stability of duplexes and triplexes. The increased affinity of formacetal oligonucleotides to complementary RNA fragments can be further increased by substituting the 3′-bridging oxygen with a sulfur atom when forming a thioacetal linkage (**28**) [73]. Based on the replacement of the phosphorus atom with a sulfur atom, a variety of modified oligonucleotides with sulfamate (**29**, **30**) [74,75], sulfamide (**31**) [76,77], sulfonamide (**32**) [78], sulfonic (**33**), and dimethylene sulfide (**34**) bridging groups [79], often having interesting characteristics, have been obtained. However, when creating modified polymers, amide fragments (**35**–**40**) are most often used as alternative acyclic linkages, allowing the internucleotide crosslinking to be configured in different ways [80,81,82,83].

An interesting non-phosphorus type of linear cross-linking is the methylene(methylimine) modification (**41**) [84]. This modification has many advantages, such as an achiral and neutral backbone, high affinity for RNA, and significant resistance to nucleases. Other acyclic linkages include carbamate variants (**42**–**43)**, which can be found in oligonucleotides with locked units [85] and urea derivatives (**44**) [86], although these are considered too short to adopt the preferred oligonucleotide conformation in the duplex. 

A complete replacement of the negatively charged phosphodiester bond is also possible with alternative positively charged structural motifs. The introduction of thiourea (**45**) [87] or guanidinium (**46**) [88] linkages into the structure of oligonucleotides, which are positively charged at physiological pH, leads to the formation of zwitterionic or even oligocationic backbone structures, and this significantly improves the absorption of such polymeric structures. Another positively charged modification of oligomers that touches on the internucleotide bond is the obtaining of nucleosyl amino acids (**47**), which can significantly increase the stability of the final polymers in biological media, such as blood plasma [89].

### 4.2. Cyclic Linkages

**Figure 7:**
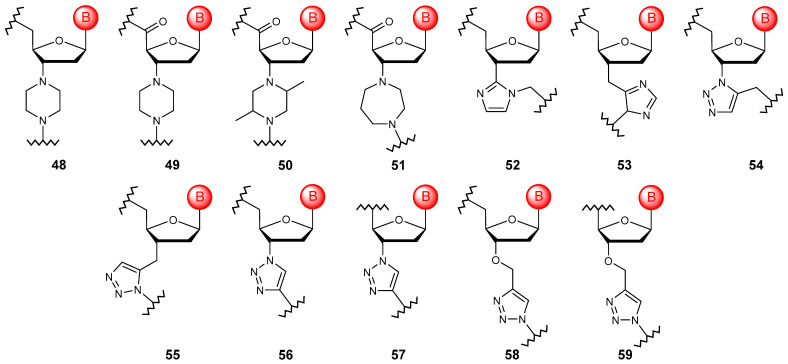
Structures of DNA monomer units with cyclic linkages (**48**–**59**).

To date, there are not many examples of cross-linking nucleoside structures into a polymer using cyclic fragments (Figure 7). However, the use of such heterocyclic systems, such as piperazine (**48**–**50**) [90], homopiperazine (**51**) [80], imidazole (**52**, **53**) [91], and triazole (**54**, **55**) [92], for these purposes can be classified as such modifications. The last modification is interesting because, with the development of click chemistry, the number of options and the ease of introducing such internucleoside cross-linking have increased significantly (**56**–**59**) [93,94]. The introduction of a heterocyclic motif into the linkage structure does not provide any benefit in the stability, binding ability, and resistance of the final polymers. However, triazole linkages are still being used, in particular, in the synthesis of oligomers from locked units [95], precisely because of the development of such a synthetic approach. Moreover, the biocompatibility of 1,5-disubstituted triazole linkages with DNA polymerases have been revealed recently [96].

## 5. Sugar Modifications

A reason for studying artificial NAs is to develop modified oligonucleotides with improved chemical and biological properties that allow them to function more efficiently than DNA/RNA in biomedical, biotechnological, and nanotechnological applications. The stability of natural oligonucleotide duplexes, as well as the formation of stable and functional protein–oligonucleotide complexes, is determined primarily by the conformation and dynamics of the sugar moiety. Thus, the ribofuranose modification in NAs is a widely used method for manipulating the activity of natural polymers.

### 5.1. Esterification of Free Hydroxy Groups in RNA

Currently, RNA-based drugs are being actively developed. However, RNA is inherently unstable, potentially immunogenic, and requires the use of special targeted delivery systems. The sensitivity of RNA to RNases is determined by the presence of the 2′-hydroxy group in the sugar ring, and therefore, modifications of the ribose fragment were primarily aimed at replacing this position.

**Figure 8:**
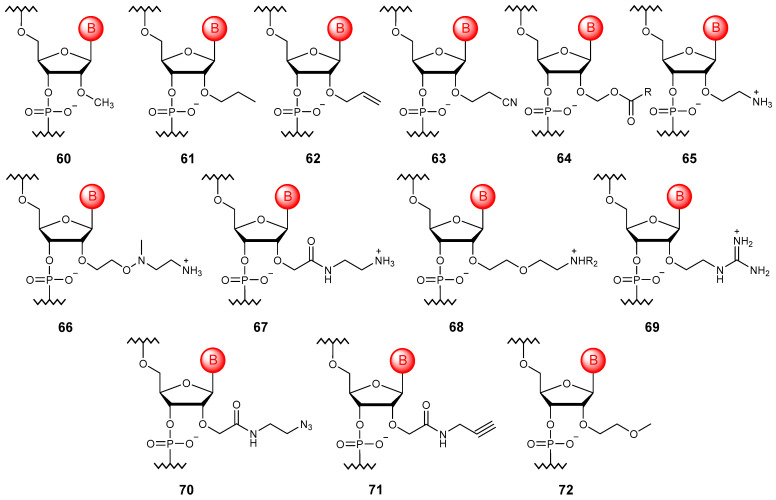
Structures of 2′-O-esterified RNA monomer units (**60**–**72**).

The simplest modification of RNA is methylation at the oxygen atom of the 2′-hydroxy group (**60**), while a similar modification occurs in nature [97]. Oligonucleotides with the 2′-O-methyl modification have increased resistance to nucleases and affinity to target RNA, as well as a reduced immune response (Figure 8). Many ether modifications of the 2′-hydroxy group have also been proposed, including alkyl modifications (**61**) [98], those containing functional groups such as 2′-O-allylic (**62**) [99], 2′-O-cyanoethyl (**63**) [100], and 2′-O-acetalester (**64**) [101], positively charged groups that could compensate for the negative charge of the phosphate residue, for example, amine (**65**–**68**) [102,103,104,105] and guanidine (**69**) [106], as well as clickable modifications (**70**, **71**) [107]. In this case, the most successful turned out to be the 2′-O-methoxyethyl modification (**72**), which allows improving therapeutically significant properties, namely increasing the affinity to RNA, resistance to nucleases, and thermal stability of the complexes [108]. This modification can be found in a number of FDA-approved oligonucleotide drugs [109].

### 5.2. The Introduction of Substituents into the Ribose Ring

**Figure 9:**
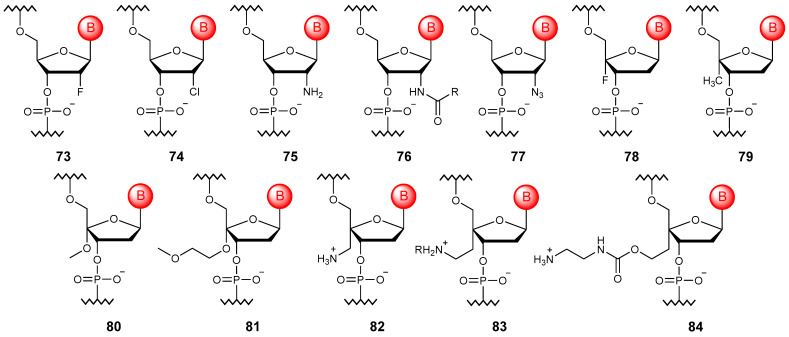
Structures of 2′- and 4′-substituted DNA monomer units (**73**–**84**).

Although the natural DNA molecule is more stable than RNA due to the absence of the 2′-hydroxy group in the ribose ring, its stability is still not sufficient for clinical use. In this regard, it was proposed to introduce substituents, primarily those with high electronegativity, into this position (Figure 9) to increase not only stability but also affinity to RNA due to conformational factors [110].

Halogen atoms, in particular, fluorine (**73**) [111] or chlorine (**74**) [112], can be introduced into the ribose ring. Such halogen-modified oligonucleotides allow one to increase resistance to nucleases while also suppressing immune stimulation. The 2′-F modification is also often used to finetune the RNA-cleaving activity of modified ASOs [113]. Electron-donating substituents, namely the amino group and its derivatives (**75**, **76**) [114], can also be introduced into the 2′ position of the ribose ring, which stimulates a more DNA-preferred configuration of ribose. In this case, the introduction of azide (**77**) [115] is the 2′ modification with the widest range of applications. Such modified oligonucleotides can be further functionalized with dyes or biotin and, like other 2′-substituted ones, be synthesized by modified DNA polymerases [112].

A number of 4′ modifications of the ribose ring have also been described in the literature; these include the introduction of fluorine (**78**) [116], methyl (**79**), alkoxy groups (**80**, **81**) [117], and amino alkyl substitutions (**82**–**84**) [118]. Oligonucleotides modified in this way represent DNA constructs that mimic the behavior of RNA. However, such modifications are most often used in combination with 2′ modifications for more precise conformational adjustments of the ribose ring [119,120].

### 5.3. Alternative Sugar Moieties

A drastic way to change the geometry and conformational mobility of the sugar moiety is to replace the ribose ring with alternative cyclic and acyclic fragments. Such modifications are aimed at modulating specific interactions and properties of oligonucleotides, including the stability and recognition ability.

#### 5.3.1. Ribose Isomers

**Figure 10:**
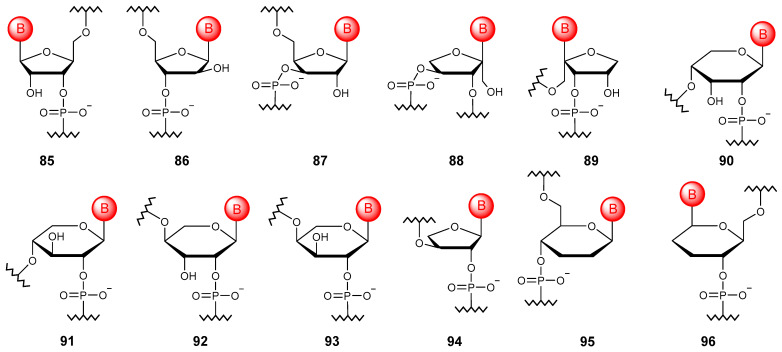
Structures of NA monomer units based on ribose isomers (**85**–**96**) and other sugars (**94**–**96**).

The natural structure of RNA is formed by β-D-ribofuranose, a cyclic form of D-ribose. If the enantiomer is used instead, that is, L-ribose (Figure 10), then the so-called spiegelmers (**85**) could be obtained. Such oligomers are non-immunogenic and practically resistant to enzymatic degradation; they do not interact with natural NAs but are widely used as aptamers [121]. It is also possible to obtain oligomers based on other isomers of ribose in the furanose form, including arabinose (**86**) [122] and xylose (**87**) [123], while the latter has an orthogonal pairing system. 

Oligonucleotide chains can also be constructed using isomeric ketopentoses. Thus, nucleic acids based on α-L-xylulofuranose (**88**) and β-L-ribulofuranose (**89**), although they do not exhibit pairing ability, but the point introduction of such elementary units allows one to enhance the orthogonal base pairing properties of the chimeric oligonucleotide [124].

If we consider the pyranose form, it is possible to obtain oligonucleotides based on ribose (**90**), xylose (**91**), lyxose (**92**), and arabinose (**93**), which have pairing abilities [125].

#### 5.3.2. Other Sugars

Other sugars such as threose and glucose can be used to construct the oligonucleotide chain (Figure 10). The threose NA (**94**) obtained in the first case is the simplest of all potential alternatives to natural NAs of the oligonucleotide type, capable of pairing with DNA and RNA and having greater hydrolytic stability compared with RNA [126]. Homo-DNA derived from 2′,3′-dideoxy-β-D-glucopyranose (**95**) does not tend to form helical structures [127], while α-homo-DNA (**96**) can interact with RNA to form parallel duplexes [128].

### 5.4. Bridged Ribose Ring Modifications

**Figure 11:**
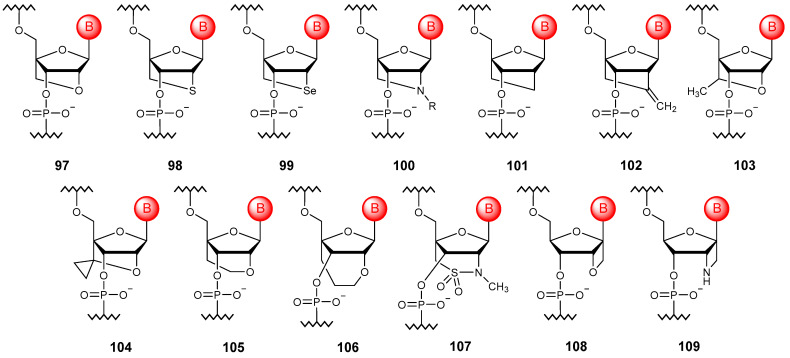
Structures of bridged NA monomer units (**97**–**109**).

To increase the affinity to RNA, a modification of the ribose ring consisting of a methylene bridge between 2′-oxygen and 4′-carbon (Figure 11) was proposed by two independent scientific groups [129,130]. This new type of oligonucleotide was called locked nucleic acids (**97**). The main idea of such a modification is to reduce the conformational mobility of ribose and increase the local organization of the carbohydrate–phosphate backbone, which leads to the formation of stronger duplexes with DNA and RNA [131,132]. 

To date, various oligonucleotide modifications that form a 2′,4′-bridge of different lengths and compositions have been proposed; these are usually referred to as 2′,4′-bridged NAs. In comparison with the initially proposed structure, the linking chain of which consisted of two atoms, carbon and oxygen, oligonucleotides with an atom of sulfur (**98**) [133], selenium (**99**) [134], nitrogen [135], including a substituted one (**100**) [136,137,138,139,140], and also simply carbon (**101**) [141] and methylene substituted carbon (**102**) [142] instead of the original heteroatom were developed. In addition, modifications with a substituent (**103**) [143] and a heterocyclic fragment (**104**) [144] within the bridge, as well as oligonucleotides with three-atom (**105**) [145] and four-atom bridged modifications (**106**) [146], including with various heteroatoms (**107**) [147], have been obtained. The whole variety of structures of such modified NAs obtained to date can be found in the review [148].

A 1′,2′-bridging modification (**108**), allowing conformational restriction of the ribose ring, has also been described in the literature [149]. Although it does not offer significant advantages over unmodified oligonucleotides, further replacement of the 2′-oxygen atom with nitrogen (**109**) allows one to increase the affinity of NAs modified in this way to RNA [150].

### 5.5. Substitution with Non-Sugar Cycles

#### 5.5.1. Monocyclic Substitution

**Figure 12:**
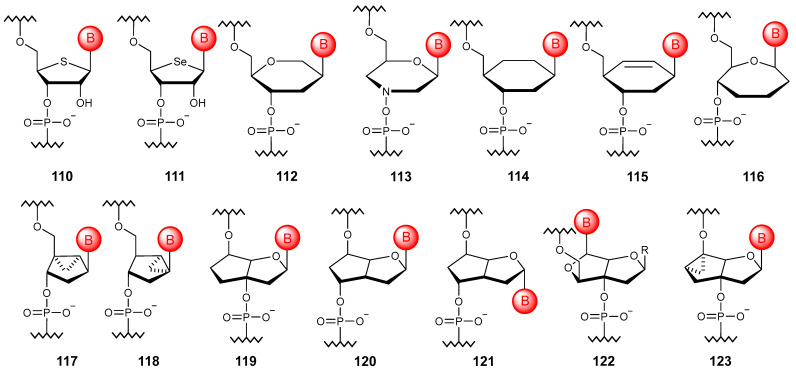
Structures of NA monomer units based on alternative cyclic motifs (**110**–**123**).

The formal replacement of the ring oxygen atom with sulfur or selenium (Figure 12) allows one to obtain the so-called thioRNA (**110**) [151] and selenoRNA (**111**) [152]. Oligonucleotides built on such artificial heterocyclic fragments have an increased ability to form duplexes with RNA and high resistance to endonucleases, despite the presence of 2′-OH groups. Another cyclic system widely used to construct the oligonucleotide chain is 1,5-anhydrohexitol. Despite the fact that all four isomers have been synthesized to date, the most studied and practically used are β-D-hexitol nucleic acids (**112**), which have a pronounced ability to hybridize with natural NAs, especially with RNA [153]. If the ribose ring is replaced by a morpholine system, then the nucleotides modified in this way are morpholino nucleic acids (**113**) [154]. 

The transition to a six-membered all-carbon scaffold was carried out in cyclohexanyl NAs, among which only the D-isomer (**114**) is capable of hybridizing with natural NAs [155]. An unsaturated modification of such oligonucleotides, called cyclohexenyl NAs (**115**), has the ability to undergo conformational adaptation when incorporated into a natural DNA chain [156]. There are also oligonucleotides based on a seven-membered ring, such as oxepane NAs (**116**), which are able to associate with their respective complementary RNA strands [157].

#### 5.5.2. Bicyclic and Tricyclic Substitutions

Bicyclic artificial NAs include methanocarbacyclic oligonucleotides (**117**, **118**) [158]. The fact that the bicyclo[3.1.0]hexyl scaffold effectively mimics the 3′-endo structure of the sugar moiety by locking the conformation of the cyclopentane ring with cyclopropyl fragment allows the modified oligonucleotides to exhibit increased affinity to RNA and stability to nuclease degradation. Formal ring closure through 3′ and 5′ carbon atoms of the ribose ring in natural NAs allows one to obtain the so-called bicyclo-DNA (**119**) [159], which has increased affinity to complementary RNA and nuclease resistance. To date, the modification with a displaced phosphodiester linkage (**120**) [160], as well as its anomeric variant (**121**) [161], has been developed. Using formal ring closure between the 2′ oxygen atom and the 3′ carbon of the arabinose ring, bicyclic oligonucleotides (**122**) with functional groups facing the major groove were proposed [162]. There are also oligomers built on a tricyclic fragment (**123**), which show some potential in the treatment of genetic disorders [163].

### 5.6. Acyclic Substitutions

**Figure 13:**
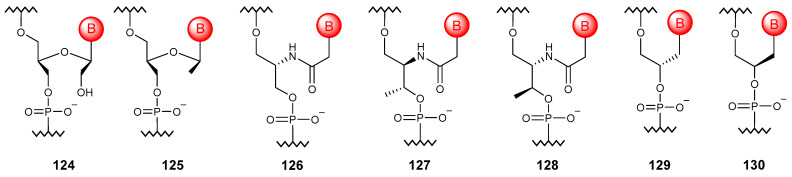
Structures of simplified NA monomer units (**124**–**130**).

The formal “cleavage” of the bond between 2′ and 3′ carbon atoms of the ribose ring produces an acyclic analog of RNA (Figure 13), unlocked NAs (**124**) capable of modulating the stability of NA duplexes and base-pairing specificity [164]. Further “cleavage” of the 2′-CH_2_OH group results in the so-called flexible NAs (**125**) that weakly hybridize with DNA [165].

If 2-amino-1,3-propanediol is used instead of ribose, it is possible to obtain serinol NAs (**126**), which form highly stable heteroduplexes with complementary DNA and RNA sequences [166]. The chirality of such oligomers depends only on the sequence, since the enantiomer is an oligomer with the reverse sequence. A nucleic acid based on L-threoninol (**127**) instead of ribose can bind to DNA and RNA, while the D-isomer (**128**) is not capable of such interactions [167]. Among the glycol NAs (**129**, **130**), only oligonucleotides in the S configuration are capable of hybridizing with RNA [168].

## 6. Alternative Scaffolds for Nitrogenous Bases

The synthetic possibilities for the formation of alternative scaffolds bearing nitrogenous bases are virtually limitless. The development and study of hybridization properties of so-called peptide NAs built both on the classic aminoethyl glycine motif [169], as well as on the basis of dipeptide structures [170] and conformationally constrained analogs [171], has outgrown into a separate research area. In addition, combinations of the modifications described above allow one to obtain new types of oligonucleotides. Along with locked NAs, morpholine-containing oligonucleotides have become mainstream in recent decades, among which phosphorodiamidate analogs are the most commercially successful and can be found in four FDA-approved drugs [109]. At the same time, a huge number of combinations have not yet been translated into reality, and perhaps some of them will lead to the emergence of unique properties of oligonucleotides that could manage diseases and pathologies for which classical drugs are ineffective.

## 7. Conclusions

Artificial NAs are an important tool for realizing the inexhaustible potential of natural NAs. Modified nucleotides have now achieved such progress that they have become an important class of therapeutic drugs. Chemical modifications are needed both to impart stability, resistance, and specificity compared with natural NAs, as well as other properties necessary for their use as drugs. This review provides basic information that allows the unprepared reader to understand the variety of possible modifications of the sugar phosphate backbone of natural NAs.

The proposed classification is not just a listing of modifications but an attempt to systematize the existing knowledge in oceanic oligonucleotide chemistry from the point of view of chemical (structural) logic. It should be noted that, in reality, it is almost impossible to find oligonucleotides based on a single modification. Most often, combinations of modifications, as well as the point inclusion of modified nucleotide units and their combinations in the oligonucleotide, are used. When designing new oligonucleotides, it is necessary to focus on modern achievements but be based on principal modifications, which will introduce a rational grain into the process of obtaining the desired characteristics. We believe that the classification presented in this review, accompanied by appropriate visualization, will inspire researchers to create oligonucleotides with new types of modifications or an original combination of known modifications, which, in the future, can become promising drugs or diagnostic tools.

## Data Availability

Not applicable.

## References

[1] Breaker R.R. (2004). Natural and engineered nucleic acids as tools to explore biology. Nature.

[2] Roberts T.C., Langer R., Wood M.J.A. (2020). Advances in oligonucleotide drug delivery. Nat. Rev. Drug Discov..

[3] Ivanov G.S., Tribulovich V.G., Pestov N.B., David T.I., Amoah A.S., Korneenko T.V., Barlev N.A. (2022). Artificial genetic polymers against human pathologies. Biol. Direct.

[4] Friedrich M., Aigner A. (2022). Therapeutic siRNA: State-of-the-art and future perspectives. BioDrugs.

[5] Keefe A.D., Pai S., Ellington A. (2010). Aptamers as therapeutics. Nat. Rev. Drug. Discov..

[6] Salem A.K., Weiner G.J. (2009). CpG oligonucleotides as immunotherapeutic adjuvants: Innovative applications and delivery strategies. Adv. Drug. Deliv. Rev..

[7] Hendel A., Bak R.O., Clark J.T., Kennedy A.B., Ryan D.E., Roy S., Steinfeld I., Lunstad B.D., Kaiser R.J., Wilkens A.B. (2015). Chemically modified guide RNAs enhance CRISPR-Cas genome editing in human primary cells. Nat. Biotechnol..

[8] Cromwell C.R., Sung K., Park J., Krysler A.R., Jovel J., Kim S.K., Hubbard B.P. (2018). Incorporation of bridged nucleic acids into CRISPR RNAs improves Cas9 endonuclease specificity. Nat. Commun..

[9] Gheibi-Hayat S.M., Jamialahmadi K. (2021). Antisense oligonucleotide (AS-ODN) technology: Principle, mechanism and challenges. Biotechnol. Appl. Biochem..

[10] Dhuri K., Bechtold C., Quijano E., Pham H., Gupta A., Vikram A., Bahal R. (2020). Antisense oligonucleotides: An emerging area in drug discovery and development. J. Clin. Med..

[11] McCown P.J., Ruszkowska A., Kunkler C.N., Breger K., Hulewicz J.P., Wang M.C., Springer N.A., Brown J.A. (2020). Naturally occurring modified ribonucleosides. Wiley Interdiscip. Rev. RNA.

[12] Lopez-Tena M., Chen S.-K., Winssinger N. (2023). Supernatural: Artificial nucleobases and backbones to program hybridization-based assemblies and circuits. Bioconjugate Chem..

[13] Lee K.H., Hamashima K., Kimoto M., Hirao I. (2018). Genetic alphabet expansion biotechnology by creating unnatural base pairs. Curr. Opin. Biotechnol..

[14] Martin A.R., Mohanan K., Luvino D., Floquet N., Baraguey C., Smietana M., Vasseur J.-J. (2009). Expanding the borononucleotide family: Synthesis of borono-analogues of dCMP, dGMP and dAMP. Org. Biomol. Chem..

[15] Miller P.S., Barrett J.C., Ts’o P.O.P. (1974). Alkyl phosphotriesters of dinucleotides and oligonucleotides. 4. Synthesis of oligodeoxyribonucleotide ethyl phosphotriesters and their specific complex formation with transfer ribonucleic acid. Biochemistry.

[16] Miller P.S., Fang K.N., Kondo N.S., Ts’o P.O.P. (1971). Conformation and interaction of dinucleoside mono- and diphosphates. V. Syntheses and properties of adenine and thymine nucleoside alkyl phosphotriesters, the neutral analogs of dinucleoside monophosphates. J. Am. Chem. Soc..

[17] Stec W.J., Zon G., Gallo K.A., Byrd R.A., Uznanski B., Guga P. (1985). Synthesis and absolute configuration of P-chiral O-isopropyl oligonucleotide triesters. Tetrahedron Lett..

[18] Letsinger R.L., Bach S.A., Eadie J.S. (1986). Effects of pendant groups at phosphorus on binding properties of d-ApA analogues. Nucleic Acids Res..

[19] Wenninger D., Hinz M., Hahner S., Hillenkamp F., Seliger H. (1998). Enzymatic and hybridization properties of oligonucleotide analogues containing novel phosphotriester internucleotide linkage. Nucleosides Nucleotides.

[20] Lancelot G., Guesnet J.L., Asseline U., Thuong N.T. (1988). NMR studies of complex formation between the modified oligonucleotide d(T*TCTGT) covalently linked to an acridine derivative and its complementary sequence d(GCACAGAA). Biochemistry.

[21] Monfregola L., Caruthers M.H. (2015). Solid-Phase Synthesis, hybridizing ability, uptake, and nuclease resistant profiles of position-selective cationic and hydrophobic phosphotriester oligonucleotides. J. Org. Chem..

[22] Tosquellas G., Alvarez K., Dell’Aquila C., Morvan F., Vasseur J.-J., Imbach J.-L., Rayner B. (1998). The pro-oligonucleotide approach: Solid phase synthesis and preliminary evaluation of model pro-dodecathymidylates. Nucleic Acids Res..

[23] Hayashi J., Samezawa Y., Ochi Y., Wada S., Urata H. (2017). Syntheses of prodrug-type phosphotriester oligonucleotides responsive to intracellular reducing environment for improvement of cell membrane permeability and nuclease resistance. Bioorg. Med. Chem. Lett..

[24] Sugimoto N., Hayashi J., Funaki R., Wada S., Wada F., Harada-Shiba M., Urata H. (2023). Prodrug-type phosphotriester oligonucleotides with linear disulfide promoieties responsive to reducing environment. ChemBioChem.

[25] Roy S., Olesiak M., Padar P., McCuen H., Caruthers M.H. (2012). Reduction of metal ions by boranephosphonate DNA. Org. Biomol. Chem..

[26] Heinonen P., Lönnberg H. (2004). Synthesis of phosphate-branched oligonucleotides. Bioconjugate Chem..

[27] Jahns H., Roos M., Imig J., Baumann F., Wang Y., Gilmour R., Hall J. (2015). Stereochemical bias introduced during RNA synthesis modulates the activity of phosphorothioate siRNAs. Nat. Commun..

[28] Tram K., Wang X., Yan H. (2007). Facile synthesis of oligonucleotide phosphoroselenoates. Org. Lett..

[29] Liang C., Allen L.C. (1987). Sulfur does not form double bonds in phosphorothioate anions. J. Am. Chem. Soc..

[30] Rahman S.M.A., Baba T., Kodama T., Islam M.A., Obika S. (2012). Hybridizing ability and nuclease resistance profile of backbone modified cationic phosphorothioate oligonucleotides. Bioorg. Med. Chem..

[31] Knouse K.W., de Gruyter J.N., Schmidt M.A., Zheng B., Vantourout J.C., Kingston C., Mercer S.E., Mcdonald I.M., Olson R.E., Zhu Y. (2018). Unlocking P(V): Reagents for chiral phosphorothioate synthesis. Science.

[32] Iwamoto N., Butler D.C.D., Svrzikapa N., Mohapatra S., Zlatev I., Sah D.W.Y., Meena, Standley S.M., Lu G., Apponi L.H. (2017). Control of phosphorothioate stereochemistry substantially increases the efficacy of antisense oligonucleotides. Nat. Biotechnol..

[33] Kamaike K., Hirose K., Kayama Y., Kawashima E. (2006). Synthesis of oligonucleoside phosphorodithioates on a solid support by the H-phosphonothioate method. Tetrahedron.

[34] Subach M.F., Khrenova M.G., Zvereva M.I. (2024). Modern methods of aptamer chemical modification and principles of aptamer library selection. Mosc. Univ. Chem. Bull..

[35] Xu D., Rivas-Bascón N., Padial N.M., Knouse K.W., Zheng B., Vantourout J.C., Schmidt M.A., Eastgate M.D., Baran P.S. (2020). Enantiodivergent formation of C–P bonds: Synthesis of P-chiral phosphines and methyl-phosphonate oligonucleotides. J. Am. Chem. Soc..

[36] Arangundy-Franklin S., Taylor A.I., Porebski B.T., Genna V., Peak-Chew S., Vaisman A., Woodgate R., Orozco M., Holliger P. (2019). A synthetic genetic polymer with an uncharged backbone chemistry based on alkyl phosphonate nucleic acids. Nat. Chem..

[37] Fathi R., Huang Q., Syi J.L., Delaney W., Cook A.F. (1994). (Aminomethyl)phosphonate derivatives of oligonucleotides. Bioconjugate Chem..

[38] Fathi R., Huang Q., Coppola G., Delaney W., Teasdale R., Krieg A.M., Cook A.F. (1994). Oligonucleotides with novel, cationic backbone substituents: Aminoethylphosphonates. Nucleic Acids Res..

[39] Sheehan D., Lunstad B., Yamada C.M., Stell B.G., Caruthers M.H., Dellinger D.J. (2003). Biochemical properties of phosphonoacetate and thiophosphonoacetate oligodeoxyribonucleotides. Nucleic Acids Res..

[40] Yamada C.M., Dellinger D.J., Caruthers M.H. (2006). Synthesis and biochemical evaluation of phosphonoformate oligodeoxyribonucleotides. J. Am. Chem. Soc..

[41] Mag M., Muth J., Jahn K., Peyman A., Kretzschmar G., Engels J.W., Uhlmann E. (1997). Synthesis and binding properties of oligodeoxynucleotides containing phenylphosphon(othio)ate linkages. Bioorg. Med. Chem..

[42] Zmudzka K., Johansson T., Wojcik M., Janicka M., Nowak M., Stawinski J., Nawrot B. (2003). Novel DNA analogues with 2-, 3- and 4-pyridylphosphonate internucleotide bonds: Synthesis and hybridization properties. New J. Chem..

[43] Krishna H., Caruthers M.H. (2012). Alkynyl phosphonate DNA: A versatile “click”able backbone for DNA-based biological applications. J. Am. Chem. Soc..

[44] Jager A., Levy M.J., Hecht S.M. (1988). Oligonucleotide N-alkylphosphoramidates: Synthesis and binding to polynucleotides. Biochemistry.

[45] Kupryushkin M.S., Zharkov T.D., Ilina E.S., Markov O.V., Kochetkova A.S., Akhmetova M.M., Lomzov A.A., Pyshnyi D.V., Lavrik O.I., Khodyreva S.N. (2021). Triazinylamidophosphate oligonucleotides: Synthesis and study of their interaction with cells and DNA-binding proteins. Russ. J. Bioorganic Chem..

[46] Michel T., Martinand-Mari C., Debart F., Lebleu B., Robbins I., Vasseur J.-J. (2003). Cationic phosphoramidate-oligonucleotides efficiently target single-stranded DNA and RNA and inhibit hepatitis C virus IRES-mediated translation. Nucleic Acids Res..

[47] Deglane G., Abes S., Michel T., Prévot P., Vives E., Debart F., Barvik I., Lebleu B., Vasseur J.-J. (2006). Impact of the guanidinium group on hybridization and cellular uptake of cationic oligonucleotides. ChemBioChem.

[48] Kupryushkin M.S., Pyshnyi D.V., Stetsenko D.A. (2014). Phosphoryl guanidines: A new type of nucleic acid analogues. Acta Naturae.

[49] Monian P., Shivalila C., Lu G., Shimizu M., Boulay D., Bussow K., Byrne M., Bezigian A., Chatterjee A., Chew D. (2022). Endogenous ADAR-mediated RNA editing in non-human primates using stereopure chemically modified oligonucleotides. Nat. Biotechnol..

[50] Chelobanov B.P., Burakova E.A., Prokhorova D.V., Fokina A.A., Stetsenko D.A. (2017). New oligodeoxynucleotide derivatives containing N-(methanesulfonyl)-phosphoramidate (mesyl phosphoramidate) internucleotide group. Russ. J. Bioorg. Chem..

[51] Patutina O.A., Gaponova Miroshnichenko S.K., Sen’kova A.V., Savin I.A., Gladkikh D.V., Burakova E.A., Fokina A.A., Maslov M.A., Shmendel’ E.V., Wood M.J.A. (2020). Mesyl phosphoramidate backbone modified antisense oligonucleotides targeting miR-21 with enhanced in vivo therapeutic potency. Proc. Natl. Acad. Sci. USA.

[52] Hara R.I., Saito T., Kogure T., Hamamura Y., Uchiyama N., Nukaga Y., Iwamoto N., Wada T. (2019). Stereocontrolled synthesis of boranophosphate DNA by an oxazaphospholidine approach and evaluation of its properties. J. Org. Chem..

[53] Takahashi Y., Kakuta K., Namioka Y., Igarashi A., Sakamoto T., Hara R.I., Sato K., Wada T. (2023). Synthesis of P-Modified DNA from boranophosphate DNA as a precursor via acyl phosphite intermediates. J. Org. Chem..

[54] Conlon P.F., Eguaogie O., Wilson J.J., Sweet J.S.T., Steinhoegl J., Englert K., Hancox O.G.A., Law C.J., Allman S.A., Tucker J.H.R. (2019). Solid-phase synthesis and structural characterisation of phosphoroselenolate-modified DNA: A backbone analogue which does not impose conformational bias and facilitates SAD X-ray crystallography. Chem Sci..

[55] Duschmalé J., Hansen H.F., Duschmalé M., Koller E., Albaek N., Møller M.R., Jensen K., Koch T., Wengel J., Bleicher K. (2020). In vitro and in vivo properties of therapeutic oligonucleotides containing non-chiral 3′ and 5′ thiophosphate linkages. Nucleic Acids Res..

[56] Barsky D., Colvin M.E., Zon G., Gryaznov S.M. (1997). Hydration effects on the duplex stability of phosphoramidate DNA-RNA oligomers. Nucleic Acids Res..

[57] Ding D., Gryaznov S.M., Wilson W.D. (1998). NMR solution structure of the N3′ --> P5′ phosphoramidate duplex d(CGCGAATTCGCG)2 by the iterative relaxation matrix approach. Biochemistry.

[58] Lelyveld V.S., Zhang W., Szostak J.W. (2020). Synthesis of phosphoramidate-linked DNA by a modified DNA polymerase. Proc. Natl. Acad. Sci. USA.

[59] An H., Wang T., Maier M.A., Manoharan M., Ross B.S., Cook P.D. (2001). Synthesis of novel 3′-*C*-methylene thymidine and 5-methyluridine/cytidine H-phosphonates and phosphonamidites for new backbone modification of oligonucleotides. J. Org. Chem..

[60] Szabó T., Kers A., Stawinski J. (1995). A new approach to the synthesis of the 5′-deoxy-5′-methylphosphonate linked thymidine oligonucleotide analogues. Nucleic Acids Res..

[61] Hutter D., Blaettler M.O., Benner S.A. (2002). From phosphate to bis(methylene) sulfone: Non-ionic backbone linkers in DNA. Helv. Chim. Acta.

[62] Parmar R., Willoughby J.L., Liu J., Foster D.J., Brigham B., Theile C.S., Charisse K., Akinc A., Guidry E., Pei Y. (2016). 5′-(E)-Vinylphosphonate: A stable phosphate mimic can improve the RNAi activity of siRNA-GalNAc conjugates. ChemBioChem.

[63] Horiba M., Yamaguchi T., Obika S. (2020). Synthesis and properties of oligonucleotides having ethynylphosphonate linkages. J. Org. Chem..

[64] Dikmen Z.G., Wright W.E., Shay J.W., Gryaznov S.M. (2008). Telomerase targeted oligonucleotide thio-phosphoramidates in T24-luc bladder cancer cells. J. Cell. Biochem..

[65] Páv O., Košiová I., Barvík I., Pohl R., Buděšínský M., Rosenberg I. (2011). Synthesis of oligoribonucleotides with phosphonate-modified linkages. Org. Biomol. Chem..

[66] Sipova H., Springer T., Rejman D., Simak O., Petrova M., Novak P., Rosenbergova S., Pav O., Liboska R., Barvik I. (2014). 5′-O-Methylphosphonate nucleic acids—New modified DNAs that increase the *Escherichia coli* RNase H cleavage rate of hybrid duplexes. Nucleic Acids Res..

[67] Ahmadibeni Y., Parang K. (2007). Synthesis and evaluation of modified oligodeoxynucleotides containing diphosphodiester internucleotide linkages. Angew. Chem..

[68] Saha A.K., Waychunas C., Caulfield T.J., Upson D.A., Hobbs C., Yawman A.M. (1995). 5′-Methyl-DNA—A new oligonucleotide analog: Synthesis and biochemical properties. J. Org. Chem..

[69] Seth P.P., Allerson C.R., Siwkowski A., Vasquez G., Berdeja A., Migawa M.T., Gaus H., Prakash T.P., Bhat B., Swayze E.E. (2010). Configuration of the 5′-methyl group modulates the biophysical and biological properties of locked nucleic acid (LNA) oligonucleotides. J. Med. Chem..

[70] Prakash T.P., Lima W.F., Murray H.M., Li W., Kinberger G.A., Chappell A.E., Gaus H., Seth P.P., Bhat B., Crooke S.T. (2015). Identification of metabolically stable 5′-phosphate analogs that support single-stranded siRNA activity. Nucleic Acids Res..

[71] Králíková Š., Buděšínský M., Rosenberg I. (2003). α-Hydroxyphosphonate oligonucleotides: A promising DNA type?. Nucleos. Nucleot. Nucl..

[72] Rozners E., Katkevica D., Strömberg R. (2007). Oligoribonucleotide analogues containing a mixed backbone of phosphodiester and formacetal internucleoside linkages, together with vicinal 2′-O-methyl groups. ChemBioChem.

[73] Zhang J., Shaw J.T., Matteucci M.D. (1999). Synthesis and hybridization property of an oligonucleotide containing a 3′-thioformcetal linked pentathymidylate. Bioorg. Med. Chem. Lett..

[74] Fettes K.J., Howard N., Hickman D.T., Adah S., Player M.R., Torrence P.F., Micklefield J. (2002). Synthesis and nucleic-acid-binding properties of sulfamide- and 3′-N-sulfamate-modified DNA. J. Chem. Soc. Perkin Trans. 1.

[75] Edward M.H., Mindy R.K., George L.T. (1992). Oligonucleotides with a nuclease-resistant sulfur-based linkage. J. Org. Chem..

[76] Micklefield J., Fettes K.J. (1998). Sulfamide replacement of the phosphodiester linkage in dinucleotides: Synthesis and conformational analysis. Tetrahedron.

[77] Kurt B.Z., Dhara D., El-Sagheer A.H., Brown T. (2024). Synthesis and properties of oligonucleotides containing LNA-sulfamate and sulfamide backbone linkages. Org. Lett..

[78] Korotkovs V., Reichenbach L.F., Pescheteau C., Burley G.A., Liskamp R.M.J. (2019). Molecular construction of sulfonamide antisense oligonucleotides. J. Org. Chem..

[79] Huang Z., Benner S.A. (2002). Oligodeoxyribonucleotide analogues with bridging dimethylene sulfide, sulfoxide, and sulfone groups. Toward a second-generation model of nucleic acid structure. J. Org. Chem..

[80] Viswanadham G., Petersen G.V., Wengel J. (1996). Incorporation of amide linked thymidine dimers into oligodeoxynucleotides. Bioorg. Med. Chem. Lett..

[81] Rozners E., Katkevica D., Bizdena E., Strömberg R. (2003). Synthesis and properties of RNA analogues having amides as interuridine linkages at selected positions. J. Am. Chem. Soc..

[82] Tanui P., Kennedy S.D., Lunstad B.D., Haas A., Leake D., Rozners E. (2014). Synthesis, biophysical studies and RNA interference activity of RNA having three consecutive amide linkages. Org. Biomol. Chem..

[83] Baker Y.R., Thorpe C., Chen J., Poller L.M., Cox L., Kumar P., Lim W.F., Lie L., McClorey G., Epple S. (2022). An LNA-amide modification that enhances the cell uptake and activity of phosphorothioate exon-skipping oligonucleotides. Nat. Commun..

[84] Morvan F., Sanghvi Y.S., Perbost M., Vasseur J.J., Bellon L. (1996). Oligonucleotide mimics for antisense therapeutics: Solution phase and automated solid-support synthesis of MMI linked oligomers. J. Am. Chem. Soc..

[85] Thorpe C., Epple S., Woods B., El-Sagheer A.H., Brown T. (2019). Synthesis and biophysical properties of carbamate-locked nucleic acid (LNA) oligonucleotides with potential antisense applications. Org. Biomol. Chem..

[86] Waldner A., Demesmaeker A., Lebreton J., Fritsch V., Wolf R.M. (1994). Ureas as backbone replacements for the phosphodiester linkage in oligonucleotides. Synlett.

[87] Arya D.P., Bruice T.C. (2000). Solid-phase synthesis of oligomeric deoxynucleic-thiourea (DNT) and deoxynucleic S-methylthiourea (DNmt): A neutral/polycationic analogue of DNA. Bioorg. Med. Chem. Lett..

[88] Blaskó A., Dempcy R.O., Minyat E.E., Bruice T.C. (1996). Association of Short-Strand DNA Oligomers with Guanidinium-Linked Nucleosides. A Kinetic and Thermodynamic Study. J. Am. Chem. Soc..

[89] Meng M., Schmidtgall B., Ducho C. (2018). Enhanced stability of DNA oligonucleotides with partially zwitterionic backbone structures in biological media. Molecules.

[90] Petersen G.V., Wengel J. (1995). Synthesis and characterization of short oligonucleotide segments containing nonnatural internucleoside amine- and amide linkages. Nucleosides Nucleotides Nucleic Acids.

[91] Beban M., Miller P.S. (2000). Preparation of an imidazole-conjugated oligonucleotide. Bioconjugate Chem..

[92] Matt P.V., Altmann K.-H. (1997). Replacement of the phosphodiester linkage in oligonucleotides by heterocycles: The effect of triazole- and imidazole-modified backbones on DNA/RNA duplex stability. Bioorg. Med. Chem. Lett..

[93] Nuzzi A., Massi A., Dondoni A. (2007). Model studies toward the synthesis of thymidine oligonucleotides with triazole internucleosidic linkages via iterative Cu(I)-promoted azide–alkyne ligation chemistry. QSAR Comb. Sci..

[94] Sanzone A.P., El-Sagheer A.H., Brown T., Tavassoli A. (2012). Assessing the biocompatibility of click-linked DNA in *Escherichia coli*. Nucleic Acids Res..

[95] Kumar P., El-Sagheer A.H., Truong L., Brown T. (2017). Locked nucleic acid (LNA) enhances binding affinity of triazole-linked DNA towards RNA. Chem. Commun..

[96] Epple S., Modi A., Baker Y.R., Wȩgrzyn E., Traore D., Wanat P., Tyburn A.E.S., Shivalingam A., Taemaitree L., El-Sagheer A.H. (2021). A new 1,5-disubstituted triazole DNA backbone mimic with enhanced polymerase compatibility. J. Am. Chem. Soc..

[97] Motorin Y., Helm M. (2011). RNA nucleotide methylation. Wiley Interdiscip. Rev. RNA.

[98] Sabahi A., Guidry J., Inamati G.B., Manoharan M., Wittung-Stafshede P. (2001). Hybridization of 2′-ribose modified mixed-sequence oligonucleotides: Thermodynamic and kinetic studies. Nucleic Acids Res..

[99] Odadzic D., Bramsen J.B., Smicius R., Bus C., Kjems J., Engels J.W. (2008). Synthesis of 2′-O-modified adenosine building blocks and application for RNA interference. Bioorg. Med. Chem..

[100] Saneyoshi H., Seio K., Sekine M. (2005). A general method for the synthesis of 2′-O-cyanoethylated oligoribonucleotides having promising hybridization affinity for DNA and RNA and enhanced nuclease resistance. J. Org. Chem..

[101] Martin A.R., Lavergne T., Vasseur J.-J., Debart F. (2009). Assessment of new 2′-O-acetalester protecting groups for regular RNA synthesis and original 2′-modified proRNA. Bioorg. Med. Chem. Lett..

[102] Odadzic D., Engels J.W. (2007). Different strategies for the synthesis of 2′-O-aminoethyl adenosine building blocks. Nucleos. Nucleot. Nucl..

[103] Prakash T.P., Kawasaki A.M., Lesnik E.A., Sioufi N., Manoharan M. (2003). Synthesis of 2′-O-[2-[(N,N-dialkylamino)oxy]ethyl]-modified oligonucleotides: Hybridization affinity, resistance to nuclease, and protein binding characteristics. Tetrahedron.

[104] Milton S., Honcharenko D., Rocha C.S.J., Moreno P.M.D., Edvard Smith C.I., Strömberg R. (2015). Nuclease resistant oligonucleotides with cell penetrating properties. Chem. Commun..

[105] Prhavc M., Prakash T.P., Minasov G., Cook P.D., Egli M., Manoharan M. (2003). 2′-O-[2-[2-(N,N-Dimethylamino)ethoxy]ethyl] modified oligonucleotides:  symbiosis of charge interaction factors and stereoelectronic effects. Org. Lett..

[106] Prakash T.P., Püschl A., Lesnik E., Mohan V., Tereshko V., Egli M., Manoharan M. (2004). 2′-O-[2-(Guanidinium)ethyl]-modified oligonucleotides:  stabilizing effect on duplex and triplex structures. Org. Lett..

[107] Wenska M., Milton S., Stromberg R. (2007). Clickable 2′-O-alkyladenosine building blocks. Nucleic Acids Symp. Ser..

[108] Egli M., Minasov G., Tereshko V., Pallan P.S., Teplova M., Inamati G.B., Lesnik E.A., Owens S.R., Ross B.S., Prakash T.P. (2005). Probing the influence of stereoelectronic effects on the biophysical properties of oligonucleotides: Comprehensive analysis of the RNA affinity, nuclease resistance, and crystal structure of ten 2′-O-ribonucleic acid modifications. Biochemistry.

[109] Egli M., Manoharan M. (2023). Chemistry, structure and function of approved oligonucleotide therapeutics. Nucleic Acids Res..

[110] Guschlbauer W., Jankowski K. (1980). Nucleoside conformation is determined by the electronegativity of the sugar substituent. Nucleic Acids Res..

[111] Ono T., Scalf M., Smith L.M. (1997). 2′-Fluoro modified nucleic acids: Polymerase-directed synthesis, properties and stability to analysis by matrix-assisted laser desorption/ionization mass spectrometry. Nucleic Acids Res..

[112] Chen T., Romesberg F.E. (2017). Enzymatic synthesis, amplification, and application of DNA with a functionalized backbone. Angew. Chem. Int. Ed..

[113] Danielsen M.B., Lou C., Lisowiec-Wachnicka J., Pasternak A., Jorgensen T., Wengel J. (2020). Gapmer antisense oligonucleotides containing 2′,3′-dideoxy-2′-fluoro-3′-C-hydroxymethyl-β-d-lyxofuranosyl nucleotides display site-specific RNase H cleavage and induce gene silencing. Chem. Eur. J..

[114] Pham J.W., Radhakrishnan I., Sontheimer E.J. (2004). Thermodynamic and structural characterization of 2′-nitrogen-modified RNA duplexes. Nucleic Acids Res..

[115] Fauster K., Hartl M., Santner T., Aigner M., Kreutz C., Bister K., Ennifar E., Micura R. (2012). 2′-Azido RNA, a versatile tool for chemical biology: Synthesis, X-ray structure, siRNA applications, click labeling. ACS Chem. Biol..

[116] Li Q., Chen J., Trajkovski M., Zhou Y., Fan C., Lu K., Tang P., Su X., Plavec J., Xi Z. (2020). 4′-Fluorinated RNA: Synthesis, structure, and applications as a sensitive 19F NMR probe of RNA structure and function. J. Am. Chem. Soc..

[117] Liboska R., Snášel J., Barvík I., Buděšínský M., Pohl R., Točík Z., Pav O., Rejman D., Novak P., Rosenberg I. (2011). 4′-Alkoxy oligodeoxynucleotides: A novel class of RNA mimics. Org. Biomol. Chem..

[118] Kanazaki M., Ueno Y., Shuto S., Matsuda A. (2000). Highly nuclease-resistant phosphodiester-type oligodeoxynucleotides containing 4′α-C-aminoalkylthymidines form thermally stable duplexes with DNA and RNA. A candidate for potent antisense molecules. J. Am. Chem. Soc..

[119] Malek-Adamian E., Patrascu M.B., Jana S.K., Martínez-Montero S., Moitessier N., Damha M.J. (2018). Adjusting the structure of 2′-modified nucleosides and oligonucleotides via C4′-α-F or C4′-α-OMe substitution: Synthesis and conformational analysis. J. Org. Chem..

[120] Koizumi K., Maeda Y., Kano T., Yoshida H., Sakamoto T., Yamagishi K., Ueno Y. (2018). Synthesis of 4′-C-aminoalkyl-2′-O-methyl modified RNA and their biological properties. Bioorg. Med. Chem..

[121] Vater A., Klussmann S. (2015). Turning mirror-image oligonucleotides into drugs: The evolution of Spiegelmer^®^ therapeutics. Drug Discov. Today.

[122] Watts J.K., Martín-Pintado N., Gómez-Pinto I., Schwartzentruber J., Portella G., Orozco M., González C., Damha M.J. (2010). Differential stability of 2′F-ANA*RNA and ANA*RNA hybrid duplexes: Roles of structure, pseudohydrogen bonding, hydration, ion uptake and flexibility. Nucleic Acids Res..

[123] Maiti M., Maiti M., Knies C., Dumbre S., Lescrinier E., Rosemeyer H., Ceulemans A., Herdewijn P. (2015). Xylonucleic acid: Synthesis, structure, and orthogonal pairing properties. Nucleic Acids Res..

[124] Efthymiou T., Gavette J., Stoop M., De Riccardis F., Froeyen M., Herdewyn P., Krishnamurthy R. (2018). Chimeric XNA—An unconventional design for orthogonal informational systems. Chem. Eur. J..

[125] Beier M., Reck F., Wagner T., Krishnamurthy R., Eschenmoser A. (1999). Chemical etiology of nucleic acid structure: Comparing pentopyranosyl-(2′→4′) oligonucleotides with RNA. Science.

[126] Schöning K., Scholz P., Guntha S., Wu X., Krishnamurthy R., Eschenmoser A. (2000). Chemical etiology of nucleic acid structure: The alpha-threofuranosyl-(3′-->2′) oligonucleotide system. Science.

[127] D’Alonzo D., Amato J., Schepers G., Froeyen M., Van Aerschot A., Herdewijn P., Guaragna A. (2013). Enantiomeric selection properties of β-homoDNA: Enhanced pairing for heterochiral complexes. Angew. Chem. Int. Ed. Engl..

[128] Nauwelaerts K., Lescrinier E., Herdewijn P. (2007). Structure of the α-homo-DNA:RNA duplex and the function of twist and slide to catalogue nucleic acid duplexes. Chem. Eur. J..

[129] Torigoe H., Hari Y., Sekiguchi M., Obika S., Imanishi T. (2001). 2′-O,4′-C-Methylene bridged nucleic acid modification promotes pyrimidine motif triplex DNA formation at physiological pH: Thermodynamic and kinetic studies. J. Biol. Chem..

[130] Koshkin A.A., Singh S.K., Nielsen P., Rajwanshi V.K., Kumar R., Meldgaard M., Olsen C.E., Wengel J. (1998). LNA (Locked Nucleic Acids): Synthesis of the adenine, cytosine, guanine, 5-methylcytosine, thymine and uracil bicyclonucleoside monomers, oligomerisation, and unprecedented nucleic acid recognition. Tetrahedron.

[131] Obika S., Nanbu D., Hari Y., Morio K., In Y., Ishida T., Imanishi T. (1997). Synthesis of 2′-O,4′-C-methyleneuridine and -cytidine. Novel bicyclic nucleosides having a fixed C_3_′-endo sugar puckering. Tetrahedron Lett..

[132] Singh S.K., Koshkin A.A., Wengel J., Nielsen P. (1998). LNA (locked nucleic acids): Synthesis and high-affinity nucleic acid recognition. Chem. Commun..

[133] Kumar R., Singh S.K., Koshkin A.A., Rajwanshi V.K., Meldgaard M., Wengel J. (1998). The first analogues of LNA (Locked Nucleic Acids): Phosphorothioate-LNA and 2′-thio-LNA. Bioorg. Med. Chem. Lett..

[134] Morihiro K., Kodama T., Kentefu Y.M., Veedu R.N., Obika S. (2013). Selenomethylene locked nucleic acid enables reversible hybridization in response to redox changes. Angew. Chem. Int. Ed. Engl..

[135] Sawamoto H., Arai Y., Yamakoshi S., Obika S., Kawanishi E. (2018). Synthetic method for 2′-amino-LNA bearing any of the four nucleobases via a transglycosylation reaction. Org. Lett..

[136] Danielsen M.B., Christensen N.J., Jorgensen T., Jensen K.J., Wengel J., Lou C. (2021). Polyamine-functionalized 2′-amino-LNA in oligonucleotides: Facile synthesis of new monomers and high-affinity binding towards ssDNA and dsDNA. Chem. Eur. J..

[137] Ejlersen M., Christensen N.J., Sorensen K.K., Jensen K.J., Wengel J., Lou C. (2018). Synergy of two highly specific biomolecular recognition events: Aligning an AT-hook peptide in DNA minor grooves via covalent conjugation to 2′-amino-LNA. Bioconjugate Chem..

[138] Shrestha A.R., Kotobuki Y., Hari Y., Obika S. (2014). Guanidine bridged nucleic acid (GuNA): An effect of a cationic bridged nucleic acid on DNA binding affinity. Chem. Commun..

[139] Lou C., Samuelsen S.V., Christensen N.J., Vester B., Wengel J. (2017). Oligonucleotides containing aminated 2′-amino-LNA nucleotides: Synthesis and strong binding to complementary DNA and RNA. Bioconjugate Chem..

[140] Ries A., Kumar R., Lou C., Kosbar T., Vengut-Climent E., Jorgensen T., Morales J.C., Wengel J. (2016). Synthesis and biophysical investigations of oligonucleotides containing galactose-modified DNA, LNA, and 2′-amino-LNA monomers. J. Org. Chem..

[141] Xu J., Liu Y., Dupouy C., Chattopadhyaya J. (2009). Synthesis of conformationally locked carba-LNAs through intramolecular free-radical addition to C=N. Electrostatic and steric implication of the carba-LNA substituents in the modified oligos for nuclease and thermodynamic stabilities. J. Org. Chem..

[142] Seth P.P., Allerson C.R., Berdeja A., Siwkowski A., Pallan P.S., Gaus H., Prakash T.P., Watt A.T., Egli M., Swayze E.E. (2010). An exocyclic methylene group acts as a bio-isostere of the 2′-oxygen atom in LNA. J. Am. Chem. Soc..

[143] Seth P.P., Siwkowski A., Allerson C.R., Vasquez G., Lee S., Prakash T.P., Wancewicz E.V., Witchell D., Swayze E.E. (2009). Short antisense oligonucleotides with novel 2′-4′ conformationally restricted nucleoside analogues show improved potency without increased toxicity in animals. J. Med. Chem..

[144] Yamaguchi T., Horiba M., Obika S. (2015). Synthesis and properties of 2′-O,4′-C-spirocyclopropylene bridged nucleic acid (scpBNA), an analogue of 2’,4′-BNA/LNA bearing a cyclopropane ring. Chem. Commun..

[145] Morita K., Hasegawa C., Kaneko M., Tsutsumi S., Sone J., Ishikawa T., Imanishi T., Koizumi M. (2002). 2′-O,4′-C-Ethylene-bridged nucleic acids (ENA): Highly nuclease-resistant and thermodynamically stable oligonucleotides for antisense drug. Bioorg. Med. Chem. Lett..

[146] Morita K., Takagi M., Hasegawa C., Kaneko M., Tsutsumi S., Sone J., Ishikawa T., Imanishi T., Koizumi M. (2003). Synthesis and properties of 2′-O,4′-C-ethylene-bridged nucleic acids (ENA) as effective antisense oligonucleotides. Bioorg. Med. Chem..

[147] Mitsuoka Y., Fujimura Y., Waki R., Kugimiya A., Yamamoto T., Hari Y., Obika S. (2014). Sulfonamide-bridged nucleic acid: Synthesis, high RNA selective hybridization, and high nuclease resistance. Org. Lett..

[148] Kawamoto Y., Wu Y., Takahashi Y., Takakura Y. (2023). Development of nucleic acid medicines based on chemical technology. Adv. Drug Deliv. Rev..

[149] Pradeepkumar P.I., Cheruku P., Plashkevych O., Acharya P., Gohil S., Chattopadhyaya J. (2004). Synthesis, physicochemical and biochemical studies of 1′,2′-oxetane constrained adenosine and guanosine modified oligonucleotides, and their comparison with those of the corresponding cytidine and thymidine analogues. J. Am. Chem. Soc..

[150] Honcharenko D., Varghese O.P., Plashkevych O., Barman J., Chattopadhyaya J. (2006). Synthesis and structure of novel conformationally constrained 1′,2′-azetidine-fused bicyclic pyrimidine nucleosides:  their incorporation into oligo-DNAs and thermal stability of the heteroduplexes. J. Org. Chem..

[151] Hoshika S., Inoue N., Minakawa N., Matsuda A. (2003). Investigation of physical and physiological properties of 4′-thioribonucleotide (4′-thioRNA). Nucleic Acids Res..

[152] Ota M., Takahashi H., Nogi Y., Kagotani Y., Saito-Tarashima N., Kondo J., Minakawa N. (2022). Synthesis and properties of fully-modified 4′-selenoRNA, an endonuclease-resistant RNA analog. Bioorg. Med. Chem..

[153] De Bouvere B., Kerreinans L., Hendrix C., De Winter H., Schepers G., Van Aerschot A., Herdewijn P. (1997). Hexitol nucleic acids (HNA): Synthesis and properties. Nucleosides Nucleotides.

[154] Chen S., Le B., Rahimizadeh K., Shaikh K., Mohal N., Veedu R. (2016). Synthesis of a morpholino nucleic acid (MNA)-uridine phosphoramidite, and exon skipping using MNA/2′-O-methyl mixmer antisense oligonucleotide. Molecules.

[155] Maurinsh Y., Rosemeyer H., Esnouf R., Medvedovici A., Wang J., Ceulemans G., Lescrinier E., Hendrix C., Busson R., Sandra P. (1999). Synthesis and pairing properties of oligonucleotides containing 3-hydroxy-4-hydroxymethyl-1-cyclohexanyl nucleosides. Chem. Eur. J..

[156] Wang J., Verbeure B., Luyten I., Lescrinier E., Froeyen M., Hendrix C., Rosemeyer H., Seela F., Van Aerschot A., Herdewijn P. (2000). Cyclohexene nucleic acids (CeNA):  serum stable oligonucleotides that activate RNase H and increase duplex stability with complementary RNA. J. Am. Chem. Soc..

[157] Sabatino D., Damha M.J. (2007). Oxepane nucleic acids: Synthesis, characterization, and properties of oligonucleotides bearing a seven-membered carbohydrate ring. J. Am. Chem. Soc..

[158] Maier M.A., Choi Y., Gaus H., Barchi J., Marquez V.E., Manoharan M. (2004). Synthesis and characterization of oligonucleotides containing conformationally constrained bicyclo[3.1.0]hexane pseudosugar analogs. Nucleic Acids Res..

[159] Bolli M., Trafelet H.U., Leumann C. (1996). Watson–Crick base-pairing properties of bicyclo-DNA. Nucleic Acids Res..

[160] Evéquoz D., Leumann C.J. (2017). Probing the backbone topology of DNA: Synthesis and properties of 7′,5′-bicyclo-DNA. Chem. Eur. J..

[161] Evéquoz D., Verhaart I.E.C., Vijver D., Renner W., Aartsma-Rus A., Leumann C.J. (2021). 7′,5′-alpha-bicyclo-DNA: New chemistry for oligonucleotide exon splicing modulation therapy. Nucleic Acids Res..

[162] Raunkjaer M., Sørensen M.D., Wengel J. (2005). Synthesis and thermal denaturation studies of novel 2′-O,3′-C-linked bicyclic oligonucleotides with a methoxy or a piperazino group facing the major groove of nucleic acid duplexes. Org. Biomol. Chem..

[163] Aupy P., Echevarría L., Relizani K., Goyenvalle A. (2017). The use of tricyclo-DNA oligomers for the treatment of genetic disorders. Biomedicines.

[164] Langkjær N., Pasternak A., Wengel J. (2009). UNA (unlocked nucleic acid): A flexible RNA mimic that allows engineering of nucleic acid duplex stability. Bioorg. Med. Chem..

[165] Merle Y., Bonneil E., Merle L., Sági J., Szemzö A. (1995). Acyclic oligonucleotide analogues. Int. J. Biol. Macromol..

[166] Le B.T., Murayama K., Shabanpoor F., Asanuma H., Veedu R.N. (2017). Antisense oligonucleotide modified with serinol nucleic acid (SNA) induces exon skipping in mdx myotubes. RSC Adv..

[167] Murayama K., Kashida H., Asanuma H. (2015). Acyclic L-threoninol nucleic acid (L-aTNA) with suitable structural rigidity cross-pairs with DNA and RNA. Chem. Commun..

[168] Schlegel M.K., Peritz A.E., Kittigowittana K., Zhang L., Meggers E. (2007). Duplex formation of the simplified nucleic acid GNA. ChemBioChem.

[169] Menchise V., De Simone G., Tedeschi T., Corradini R., Sforza S., Marchelli R., Capasso D., Saviano M., Pedone C. (2003). Insights into peptide nucleic acid (PNA) structural features: The crystal structure of a D-lysine-based chiral PNA-DNA duplex. Proc. Natl. Acad. Sci. USA.

[170] Bartolami E., Gilles A., Dumy P., Ulrich S. (2015). Synthesis of α-PNA containing a functionalized triazine as nucleobase analogue. Tetrahedron Lett..

[171] Suparpprom C., Vilaivan T. (2022). Perspectives on conformationally constrained peptide nucleic acid (PNA): Insights into the structural design, properties and applications. RSC Chem. Biol..

